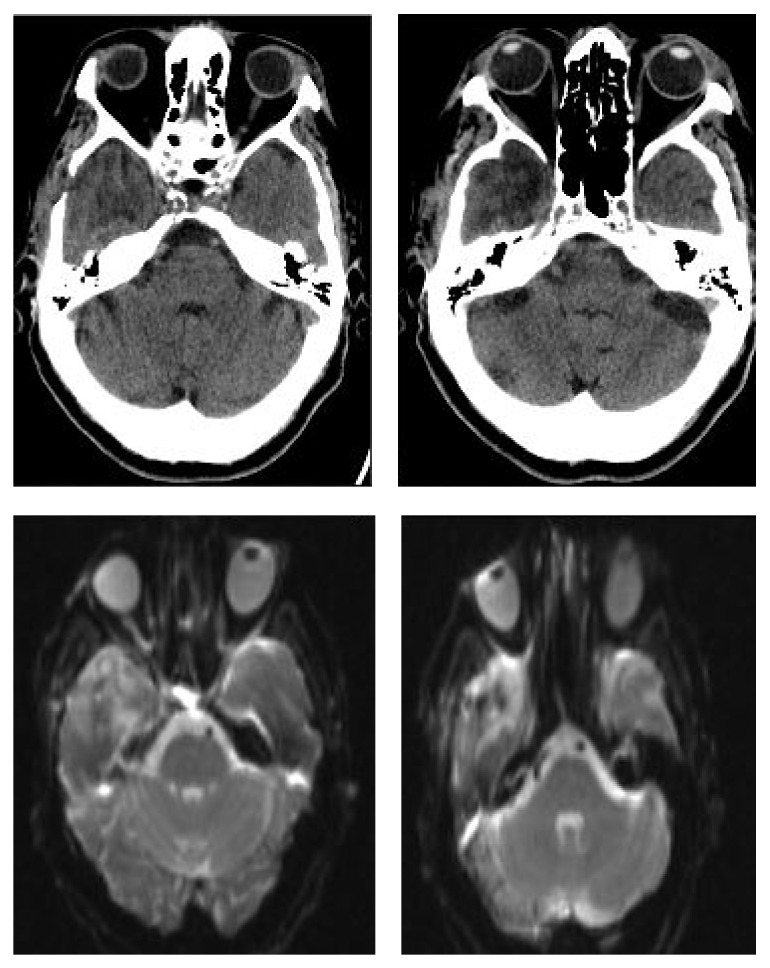# Brain Abscess after Percutaneous Therapy for Trigeminal Neuralgia

**DOI:** 10.1155/2015/162793

**Published:** 2015-03-03

**Authors:** Michele Acqui, Pietro Familiari, Alessandro Pesce, Giada Toccaceli, Antonino Raco

**Affiliations:** Neurosurgery Unit, Department of NESMOS, Faculty of Medicine and Psychology, University of Rome “La Sapienza”, Sant'Andrea Hospital, Via di Grottarossa 1035/1039, 00189 Rome, Italy

## Abstract

We report a case of brain abscess following the percutaneous treatment for trigeminal neuralgia. This procedure envisages the access with a needle into the middle cranial fossa through the oral cavity. Thus, in this case, the bacterial infection can be more likely ascribed to the possible contamination of the needle inside the oral cavity rather than to other frequent and more controllable causes of infection like an imperfect sterilization of surgical instruments or an inadequate antiseptic preparation of both operator's hands and patient's skin. The subsequent brain abscess was treated with antibiotic therapy (Vancomycin 2 gr a day and Meropenem 8 g a day for 22 days before the surgical procedure and 30 days after, until complete normalization of laboratory parameters, clinical parameters, and neurological symptoms) and surgical drainage, although the culture of the abscess capsule and the purulent material resulted sterile. In conclusion, the percutaneous therapy for trigeminal neuralgia can be objectively related to risks, even if performed by expert hands. Therefore, it is important that the patient should be advised regarding risks/benefits and/or septic complications of such procedures, even if they occur very seldom. An association of surgery and antibiotic therapy results as effective treatment for this pathologic condition.

## 1. Background 

Percutaneous radiofrequency thermocoagulation of the Gasserian ganglion, glycerol rhizotomy, and balloon compression with a balloon-tip (Fogarty) catheter are common surgical procedures against trigeminal neuralgia. These treatments are performed through the transoval percutaneous access and, however mininvasive, they can be associated to some severe or even fatal [1–3] complications such as carotid-cavernous fistulas [4–7], subdural, subarachnoid, and temporal lobe intraparenchymal hemorrhage [5, 8, 9], optic nerve lesions [10–12], deficit of the third [13, 14], fourth [5, 15], and sixth [5, 16, 17] cranial nerves.

In the recent literature, 19% of brain abscesses is related to previous neurosurgical treatment [18], but rarely following percutaneous ablation procedures for trigeminal neuralgia, representing an unusual event. A study by Kanpolat et al. [13] considered 1600 patients affected by trigeminal neuralgia and treated with radiofrequency rhizotomy, not reporting any septic complications. In 14000 cases reviewed in the literature, there are only 7 reports of meningitis and 2 of brain abscesses [3, 19, 20], representing 0.06–0.4% of total cases [3, 9, 20–25].

We report a case of trigeminal neuralgia after chemical rhizotomy with subsequent radiofrequency thermocoagulation of the Gasserian ganglion, complicated by temporal homolateral brain abscess.

## 2. Clinical Presentation

An 85-year-old woman presented a long-term history of trigeminal neuralgia (about 20 years). In addition, she had been treated with Dicumarol for chronic atrial fibrillation and she underwent surgery for ascendant aorta aneurysm, 2 aortocoronary bypasses, and valvular reconstruction.

A trigeminal glycerol rhizotomy was performed 9 years before by a surgeon expert in percutaneous therapy for trigeminal neuralgia; after a clear initial improvement, the symptoms reappeared 4 years later, leading to three new trigeminal glycerol rhizotomies (4, 5, and 8 years later), with temporary pain relief. As pain reappeared a year later, a selective thermocoagulation of the first and second right trigeminal branches was performed. Hypoaesthesia was still observed in the affected dermatomes without pain or corneal trophic complications.

The preoperative disinfection protocol admitted the use of a solution containing 10% weight by volume povidone-iodine; the antibiotic prophylaxis envisaged administration of Cefazolin (2 g before and during surgery and 1 g after). The same antibiotic was administrated for three days after surgery (3 g a day), according to our hospital's previous guidelines on antimicrobial prophylaxis in surgery (now our protocol admits Cefazolin 2 g before and during surgery, 1 g after).

**Figure 1:**
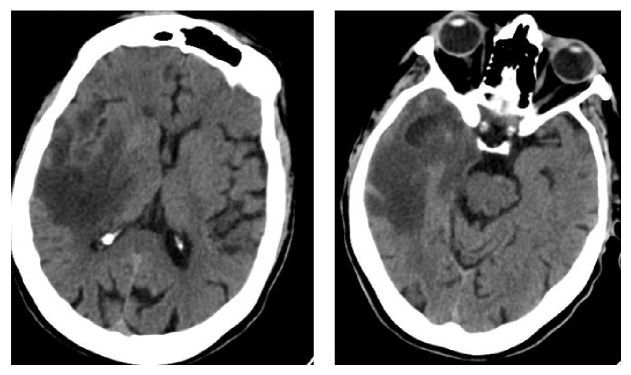


The woman was admitted in our unit due to the presence of a right frontoorbitary pressure like-headache associated with temporospatial disorientation and confabulation. The CT (Figure 1) showed a wide hypodense area located in the right temporal lobe, extending to the basal ganglia, thalamus, and posterior limb of the internal capsule. Moreover, a markedly hypodense temporopolar zone was present. The temporal lobe appeared swollen, and the right lateral ventricle was compressed with slight leftwards shifting of the supratentorial ventricular system.

**Figure 2:**
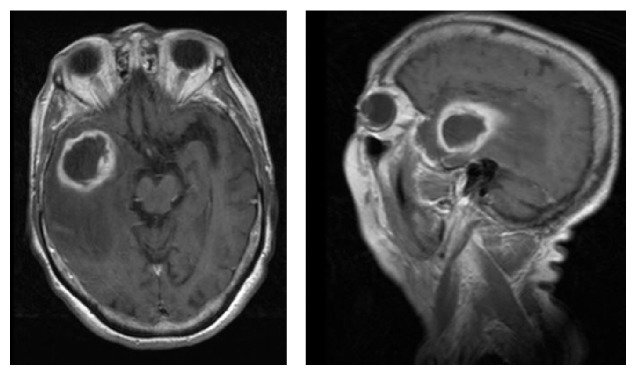


The MRI (Figure 2) evidenced a diffused signal alteration involving the right temporal lobe, the subcortical frontoparietal white matter, and the basal ganglia almost completely. In addition, the temporopolar area showed an ovoid nucleus, almost 3 cm in axial diameter, with a smaller and medial one. The lesions showed a ring enhancement and restricted diffusion (low signal on ADC). These findings on MRI suggested a right temporopolar abscess and wide vasogenic oedema, likely due to vascular congestion and stasis. Both hemispheres evidenced chronic microangiopathy of the subcortical white matter.

The patient was apyretic. Laboratory findings showed 14.11 × 10^3^/mm^3^ WBC (13.09 × 10^3^/mm^3^ N), pcr 1.2 mg/dL, and procalcitonin 0.328 ng/dL. The blood and urine cultures were negative.

**Figure 3:**
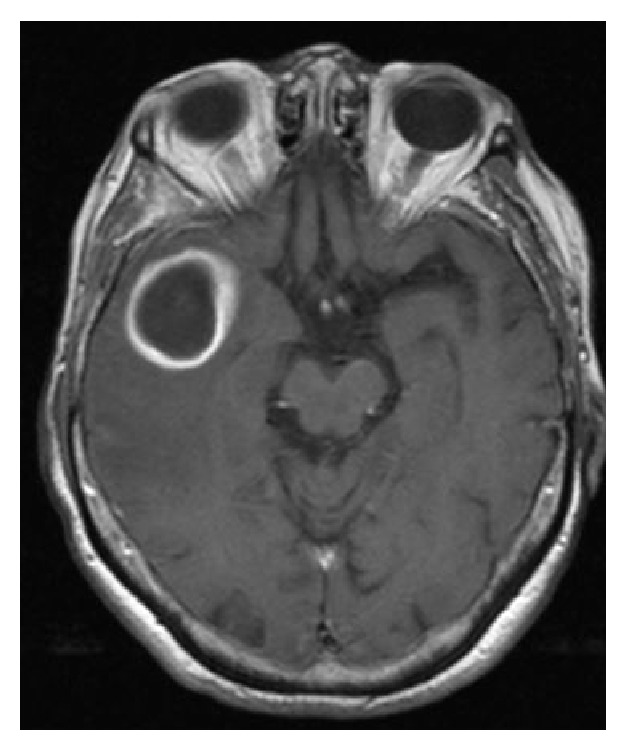


In spite of the presence of indication for surgical abscess drainage, the patient refused surgery requesting to be treated only with IV antibiotic therapy (Vancomycin 2 gr a day and Meropenem 8 g a day). After a 20-day administration of antibiotic therapy with Vancomycin 2 g a day and Meropenem 8 g a day, clinical symptoms, especially initial psychic disturbances, gradually improved, and a new MRI (Figure 3) confirmed the presence of a right temporal area with signal alterations. The lesion appeared somewhat larger than before and the surrounding oedema, as well as the related compressive effects upon the medial structures, was reduced; after i.v. administration of Gd-DTPA the ring enhancement showed a more regular shape.

**Figure 4:**
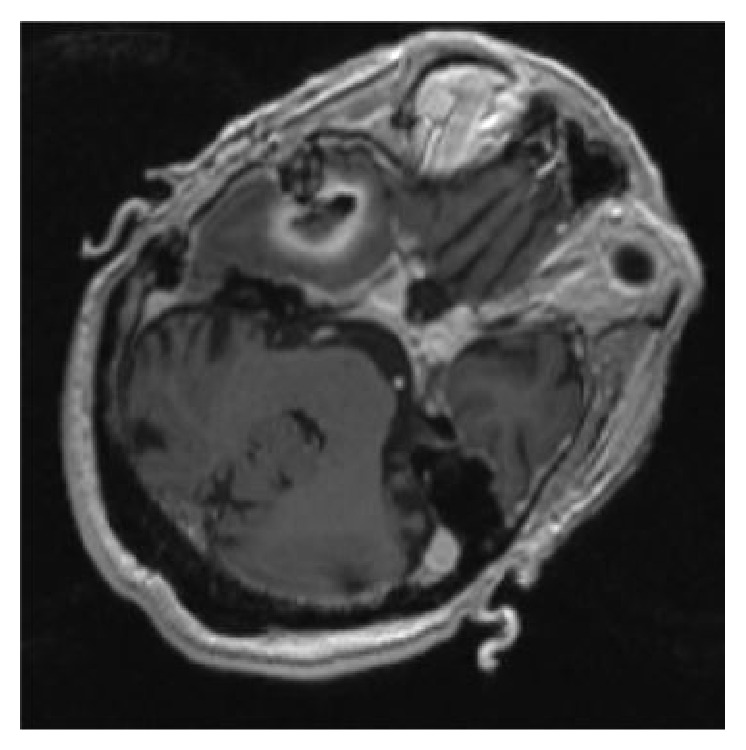


Considering particularly the volume increase of the lesion, the patient gave consent to undergo surgery and, on the twenty-second day after the beginning of the antibiotic therapy, a surgical neuronavigator-guided evacuation of the abscess was performed. Although the original surgical planning envisaged the use of a Sedan-type needle, after corticotomy the needle did not penetrate into the cavity but just dislocated it medially due to the marked fibrosis of the abscess capsule. Therefore we decided to extend the burr hole and the corticotomy and to expose the lateral portion of the abscess capsule. Once the capsule was lanced the cavity appeared full of a characteristic yellowish odourless pus. The intraoperatory MRI (Figure 4) showed the reduction of the lesion and the collapse of the abscess walls, and the absence of the restricted diffusion on DWI-MRI confirmed the correct evacuation of the purulent content. It is interesting to note that after culture the fragments of the abscess capsule and the purulent material resulted to be sterile. Also in this procedure was applied the same preoperative disinfection protocol with a solution containing 10% weight by volume povidone-iodine, while Cefazolin was not used as antibiotic prophylaxis because the patient was already being treated with Vancomycin and Meropenem.

Postoperatory evolution was absolutely normal. On the seventh day after surgery, the patient was apyretic, WBC was 8.04 × 10^3^/mm^3^ (5.03 × 10^3^/mm^3^ N), pcr was 0.9 mg/dL, and procalcitonin was 0.0662 ng/dL. The clinical situation remained stable and the patient continued precautionarily the IV antibiotic therapy with Vancomycin 2 g a day and Meropenem 8 g a day for about a month, until complete normalization of laboratory parameters (pcr, Procalcitonin and WBC), clinical parameters (patient apyretic), and neurological symptoms. No oral antibiotic therapy was administrated. A TC and MRI with DWI sequences was performed after 6 months and showed the absence of the previous radiological findings. Trigeminal pain was absent (Figure 5).

## 3. Discussion

The pathogenesis of the right temporal abscess is likely to be ascribed to the percutaneous procedures which the patient underwent. In particular, the homolaterality of the abscess and of the percutaneous procedures, the chronological sequence of its formation, and the absence of predisposing factors support an iatrogenous pathogenesis. Regarding the clinical and radiological evolution, it is likely that encephalitis and successive abscess were installed at the occasion of the last procedure that the patient was subjected to.

At the level of the Gasserian ganglion the dura mater of the middle brain fossa splits into two layers, forming the so-called dura proper, one layer facing the temporal lobe, the other fusing with the ganglionar periosteum [5]. In order for the needle to penetrate the subarachnoid space and the parenchyma of the temporal lobe, the dura proper must be perforated. Thus, when the angle is too steep, the needle tip may enter into the subarachnoid space, and even into the temporal lobe [9]. By this mean a bacterial charge can penetrate directly inside the parenchyma and cause a brain abscess.

Intracranial bacterial contamination may be caused by an imperfect sterilization of surgical instruments, lacking operator's asepsis, inadequate skin antiseptic preparation [20], or, most probably for a brain abscess, penetration of the needle inside the oral cavity [2, 19, 20]. The latter condition can be controlled by maintaining the index finger inside the oral vestibule while the needle penetrates the facial soft tissues [2, 26]. If penetration of the oral cavity occurs, the needle should be withdrawn and replaced with a sterile one, in order to avoid the introduction of oral commensals into the middle cranial fossa [20]. In such cases, postoperatory antibiotic therapy is always indicated [20]. Even though the patient's surgical history did not report penetration of the oral cavity by the needle, presence of bacteria in the submucosa and subsequent passage into the foramen ovale cannot be excluded [23].

Considering the pathogenesis, there was a good probability that the infective agent might be staphylococci. Therefore, the patient was treated empirically with Vancomycin and Meropenem. At the beginning, the chosen therapy was a nonsurgical one justified by the diagnostic reliability of neuroradiological evaluations and by the substantial negativity of the neurological objective examination [27, 28].

The decision to proceed surgically was based on volumetric increase of the abscess, which would have interfered with the efficacy of the subsequent antibiotic treatment.

## 4. Conclusions

Preoperatory clinical conditions represent one of the most relevant prognostic factors in surgical treatment of brain abscesses [27, 29, 30]. Our patient showed a gradual improvement between the first diagnosis of brain abscess and surgery, in association with the antibiotic therapy she followed. Even if she was an elderly patient, with a high cardiovascular risk, she arrived to surgery in good neurological and general conditions. This situation has certainly affected positively the subsequent clinical course.

The present case report describes rare and exceptional findings which evidence how percutaneous therapy for trigeminal neuralgia can be objectively related to risks, even if performed by expert hands. Therefore, it is important that the patient should be advised regarding risks/benefits and/or septic complications of such procedures.

## Figures and Tables

**Figure 5:**